# Seroprevalence and Risk Factors for Hepatitis B, HIV, and Syphilis Among Survivors of Sexual Violence in the Eastern Cape Province, South Africa

**DOI:** 10.3390/pathogens14030209

**Published:** 2025-02-20

**Authors:** Oladele Vincent Adeniyi, Charity Masilela, Oyewole Christopher Durojaiye

**Affiliations:** 1 Department of Family Medicine, Faculty of Medicine & Health Sciences, Walter Sisulu University, East London 5200, South Africa; oadeniyi@wsu.ac.za (O.V.A.); chemasilela@gmail.com (C.M.); 2 Department of Infection and Tropical Medicine, Sheffield Teaching Hospitals NHS Foundation Trust, Sheffield S10 2JF, UK; 3 Department of Medical Microbiology, University Hospitals of Derby and Burton NHS Foundation Trust, Derby DE22 3NE, UK

**Keywords:** gender-based violence, sexual assault, sexual violence, sexually transmitted infections, South Africa

## Abstract

Understanding the prevalence of hepatitis B (HBV), human immunodeficiency virus (HIV), and syphilis among survivors of sexual violence in South Africa is crucial for guiding targeted healthcare interventions, despite the limited available data. This study aimed to investigate the prevalence of these infections and their associated risk factors in survivors from the Eastern Cape Province, South Africa. This retrospective cross-sectional study reviewed 1957 medical records of survivors of sexual violence who received care at two large healthcare facilities in the Eastern Cape Province of South Africa between January 2019 and December 2020. All survivors were screened for HBV, HIV, and syphilis infections. Logistic regression analysis was used to identify factors associated with HIV and syphilis infections. The overall seroprevalence rates for HBV, syphilis, and HIV were 0.7%, 4.9%, and 17.3%, respectively. Six individuals were co-infected with at least two of these infections. Predictors for HIV infection included age (age < 16: adjusted odds ratio [aOR] 0.05; 95% confidence interval [CI], 0.03–0.08 and ages 16–25: aOR 0.45; 95% CI, 0.34–0.59) and black race (aOR 4.78; 95% CI, 1.09–20.88). Predictors for syphilis infection were age (age < 16: aOR 0.05; 95% CI, 0.02–0.15 and ages 16–25: aOR 0.41; 95% CI, 0.25–0.66) and residing in an urban area (aOR 0.23; 95% CI, 0.10–0.50). Survivors of sexual violence are at increased risk of HBV, HIV, and syphilis. Urgent measures are needed to provide comprehensive screening, treatment, prevention, and education to address this critical public health issue.

## 1. Introduction

Sexual violence is a grave human rights violation and a profound public health issue that can harm survivors’ physical, mental, sexual, and reproductive health while also increasing the risk of acquiring sexually transmitted infections (STIs) such as human immunodeficiency virus (HIV), hepatitis B virus (HBV), and syphilis [1]. It devastates individuals, families, and communities, perpetuating cycles of trauma, fever, and inequality while undermining social and economic development. Sexual violence includes any sexual act, any attempt to obtain a sexual act, unwanted sexual comments or advances, or other acts directed against a person’s sexuality through coercion by any individual, regardless of their relationship to the victim, in any setting [2]. It encompasses rape, attempted rape, unwanted sexual touching, and other non-contact forms [1]. Although men are also victims of sexual violence, it is more prevalent among women. Globally, about 30% of women have experienced either physical and/or sexual intimate partner violence or non-partner sexual violence [1]. Gender inequality and norms that condone violence against women have been identified as the major root causes of violence against women [3,4].

South Africa has one of the highest rates of sexual violence globally, with the country often referred to as the ‘rape capital’ of the world [5,6]. The Eastern Cape of South Africa, where this study was conducted, experiences high levels of sexual violence against women and children [7,8], which may exacerbate the sexual transmission of HIV, HBV, and syphilis in the region. Therefore, our aim was to determine the seroprevalence and factors associated with HIV, HBV, and syphilis infections among survivors of sexual violence in the province. The findings from this study could offer valuable insights for the establishment of appropriate measures to mitigate the public health impact of sexual violence and improve the overall health and well-being of affected individuals in the province and other similar settings.

## 2. Materials and Methods

### 2.1. Study Design and Setting

We conducted a retrospective cross-sectional study in two large survivor centres (Thuthuzela Care Centres) at Cecilia Makiwane and Butterworth Hospitals in the central region of the Eastern Cape, South Africa. These two care centres are strategically located to provide acute services for residents of East London and Mdantsane in Buffalo City Metropolitan Municipality, as well as Butterworth township and the surrounding rural communities of Amathole District, serving a combined population of over a million people. The centres provide multidisciplinary care for survivors (individuals who experience any form of sexual violence) seeking acute assistance. Trained counsellors first debrief the survivors before they undergo triage, including vital sign measurement by nurses. Afterward, the attending forensic nurse or doctor draws blood for HIV (enzyme-linked immunosorbent assay [ELISA], syphilis (rapid plasma reagin [RPR]/treponema pallidum haemagglutination assay [TPHA]), and hepatitis B surface antigen (HBsAg) testing. The attending doctor also conducts a physical examination and evidence collection for forensic analysis. Based on referrals, survivors may also receive services from social workers and/or psychologists as needed.

### 2.2. Data Collection

Medical records of survivors who received acute care at the two centres between 1 January 2019 and 31 December 2020 were extracted for review. A total of 1957 survivors received acute care at these two centres during the study period. Medical records of the survivors were identified, retrieved, and reviewed for information relevant to the study’s objective. A pre-designed data collection proforma based on the study’s objective was used to collect data between May and June 2022.

### 2.3. Measures

HIV, syphilis, and hepatitis B status was confirmed from the medical records and records of the National Health Laboratory Services. Blood samples are routinely drawn for these STIs as part of the acute care package for survivors. HIV ELISA, RPR/TPHA, and HBsAg test results were reviewed for survivor management and transcribed into the proforma. The following covariates were extracted and categorised: age, sex, race, and residential type. Residential type was defined as any area where people live, categorised as rural, peri-urban, and urban. Notable racial groups are categorised as black (black African ancestry), white (multiracial ethnic group of European ancestry), and other (which includes mixed ancestry and Asian). Any undocumented data in each variable were considered missing data (either not screened or missing results), highlighting areas for data quality improvement in the health facilities.

COVID-19 was declared a global pandemic by the World Health Organisation (WHO) on 11 March 2020. To better understand its impact on the prevalence of HBV, HIV, and syphilis among survivors, we analysed data based on South Africa’s lockdown restriction levels. During the restrictions, healthcare priorities shifted toward managing the COVID-19 crisis. Alert levels 1 and 2 were categorised as low restrictions, while alert levels 3 to 5 were classified as moderate to high restrictions. The lockdown periods for each alert level were as follows: 26 March to 30 April 2020 (alert level 5); 1 to 31 May 2020 (alert level 4); 1 June to 17 August 2020 (alert level 3); 18 August to 20 September 2020 (alert level 2); and 21 September to 28 December 2020 (alert level 1).

### 2.4. Statistical Analysis

Data were captured on Excel spreadsheet and cleaned. The data were then exported to IBM SPSS Statistics for Windows, Version 28.0 (IBM Corp., Armonk, NY, USA). Baseline characteristics of the survivors were analysed using descriptive statistics. Categorical data are presented as frequencies (percentages), while numerical data are expressed as means with standard deviations (SDs). The prevalence of the various STIs was estimated as the proportion of survivors with positive results in relation to the total. Multivariate logistic regression models (both crude and adjusted odds ratios with their 95% confidence intervals) were computed to assess the association between the baseline characteristics of the survivors and the prevalence of HIV and syphilis. Racial groups were re-categorised into two groups: black and other, as black Africans constituted the substantial majority. All *p*-values of less than 0.05 were considered statistically significant. To evaluate the pattern of missing data, we used Little’s MCAR (Missing Completely at Random) test, which indicated that data were not missing at random (*p* < 0.001). To further understand the impact of missing data, we compared the demographic characteristics of individuals with and without test results for HIV, HBV, and syphilis. The statistical differences observed between these groups suggested that incorporating participants with missing data into the logistic regression models would have influenced the overall findings (see Appendix A Table A1).

## 3. Results

### 3.1. Sociodemographic Characteristics and Seroprevalence of HBV, HIV, and Syphilis Infection

This study recorded 1957 cases of sexual violence. Table 1 presents the sociodemographic and clinical characteristics of the cohort. Most of the survivors were female (93.6%). The mean age of the survivors was 21.7 years (SD, 16.1), with an age range of 8 months to 98 years. Of the survivors, 23.1% were under the age of 12 years, 96.3% were black, and 45.6% resided in rural areas of the province. In addition, 18.8% (*n* = 368), 22.4% (*n* = 439), and 23.3% (*n* = 456) of the survivors were not screened for HIV, HBV, and syphilis, respectively. Those under the age of 12 years were less likely to be screened for these infections (Table 1).

Two survivors (0.1%) were found to have HBV/HIV co-infection, while another two had HBV/syphilis co-infection. In addition, two more tested positive for all three infections. Five of the six co-infections occurred in survivors over the age of 25 years.

Of the 1957 survivors, 1589 (81.2%) had an HIV test result. The seroprevalence rate of HIV in the cohort was 17.3% (338/1957). Among those over the age of 16, the prevalence rate was 29.1% (313/1074). The mean age of individuals who tested positive for HIV was 29.3 years (SD, 11.4), which was higher than those who tested negative (21.1 years; SD, 16.3). The majority of survivors who tested positive were over 25 years old (56.8%), female (97.0%), and resided in rural (46.7%) or peri-urban (46.4%) settings.

A total of 1501 (76.7%) survivors had results for syphilis testing, with 4.9% (96/1957) testing positive. The mean age of those who tested positive for syphilis was 37.6 years (SD, 18.5). Most of those who tested positive for syphilis were older than 25 years (66.7%; 64/96), female (92.5%), and resided in peri-urban settings (66.7%). Syphilis infection was not recorded in survivors under the age of 12 years.

Hepatitis B testing was performed on 1518 survivors (77.6%). The overall seroprevalence rate of HBV was 0.7% (14/1957), with a mean age of 38.3 years (SD, 22.5). The highest seroprevalence was observed in individuals over 25 years old (71.4%), female (92.9%), and those living in non-urban settings (85.7%; 12/14)

### 3.2. Factors Associated with HIV and Syphilis Infection

Table 2 presents the results of the logistic regression analysis examining the factors associated with HIV and syphilis infections. After adjusting for age and place of residence, we found that the risk of HIV infection was lower among survivors under 25 years of age (<16 years: adjusted odds ratio [aOR], 0.05; 95% confidence interval [CI], 0.03–0.08; 16–25 years: aOR, 0.42; 95% CI, 0.34–0.59). However, the risk was higher among black individuals (aOR, 4.78; 95% CI, 1.09–20.88). Similarly, the likelihood of syphilis infection was lower in survivors under 16 years of age (aOR, 0.05; 95% CI, 0.02–015) and those aged 16–25 years old (aOR, 0.41; 95% CI, 0.25–0.66). Additionally, residing in an urban area was associated with a lower likelihood of testing positive for syphilis (aOR, 0.23; 95% CI, 0.10–0.50). Due to the low prevalence of HBV (0.7%; *n* = 14) in the cohort, we did not explore its associated factors.

### 3.3. Effect of COVID-19 Pandemic

Table 3 illustrates the distribution of HIV, HBV, and syphilis cases among survivors before (January 2019 to early March 2020) and during the COVID-19 pandemic, categorised by lockdown restriction levels. The majority of cases—HIV (69.2%; 234/338), syphilis (70.8%; 68/96), and HBV (78.6%; 11/14)—were recorded in the pre-pandemic period rather than during the pandemic. Among survivors who tested positive for any of these infections during the pandemic, most cases were documented during the alert level 1 lockdown (21 September to 28 December 2020).

## 4. Discussion

This study sought to examine the prevalence and factors associated with HIV, HBV, and syphilis infections among male and female survivors of sexual violence in the Eastern Cape Province of South Africa. We observed overall seroprevalence rates of 0.7%, 4.9%, and 17.3% for HBV, syphilis, and HIV, respectively, in the cohort.

Nearly half (49%) of the survivors in the cohort were minors under the age of 18. These findings are consistent with previous local and international studies that highlight the widespread occurrence of sexual violence against children and adolescents globally [1,5,9]. Although the majority of sexual violence survivors in the cohort were women and girls, it is important to recognise that men and boys accounted for 6% of all recorded cases. While sexual violence is often perceived as primarily affecting women, men and boys are also at risk. Male survivors may face unique challenges, including stigma, shame, and reluctance to report owing to gender norms and stereotypes [10,11]. This can result in an underestimation of the true prevalence of sexual violence against males within the study population. Even though male survivors represent a relatively small proportion of overall cases, their experiences deserve serious attention.

About one-fifth of the survivors in our cohort, particularly those under the age of 12, were not screened for any of the three infections. The decision for further investigation in those under 12 years old depends on clinical evidence (history and examination) supporting oral or ano-genital penetration in this age group. As such, the attending clinician may not draw blood for these tests, whenever such is deemed unnecessary. However, opportunistic screening for these STIs should not be overlooked by clinicians. This is especially so since failure to prioritise testing for HIV and other STIs as part of routine post-assault care increases survivors’ risks of undiagnosed infections. It not only jeopardises their recovery and long-term health but hinders efforts to curb further transmission in the broader community. Addressing this issue requires increased awareness, trauma-informed care, and targeted public health interventions that ensure sexual violence survivors receive the comprehensive medical support they need, including accessible STI testing and counselling services.

Among all provinces in South Africa, the Eastern Cape has the third-highest HIV burden [12]. The HIV seroprevalence rate of 29.1% among survivors over 16 years of age in our cohort is higher than the estimated adult prevalence of 25.2% for the province [12]. Several studies have established a connection between sexual violence and HIV acquisition [13,14,15]. For example, a study in Uganda found that sexually active women aged 15–49 years who had experienced partner violence were 1.6 times more likely to contract HIV than those who had not experienced such violence [15]. Although the sex of the survivors was not associated with a higher risk of HIV infection, we observed that female survivors had a higher HIV prevalence (17.9%) than male survivors (8%). In South Africa, females bear a disproportionately higher burden of HIV than males [12,16]. It is well documented that women are two to four times more likely than men to contract the virus through unprotected vaginal intercourse [17,18]. Addressing the combined issues of sexual violence and HIV can enhance outcomes for women and contribute to a healthier, more equitable society.

The true seroprevalence of HBV infection in the province remains uncertain. However, our cohort exhibited a notably lower HBV prevalence of 0.7% compared to the national HBsAg prevalence estimates of 4.7% in 2022 [19]. The reasons for the low prevalence in our cohort are unclear. It is important to recognise that variations in HBV prevalence have been reported across sub-Saharan Africa, including South Africa, depending on the region, population studied, and diagnostic methods used [20,21]. Unsurprisingly, 71.4% of confirmed hepatitis cases occurred in survivors aged over 25 years, with a prevalence of 1.8% in this age group. This is not unexpected, as most individuals over 25 were unlikely to have received the HBV vaccine during infancy, given that it was only introduced into South Africa’s Expanded Programme of Immunisation in 1995 [22,23].

Epidemiological data on syphilis prevalence in South Africa is limited, but certain population groups, such as adolescent girls and young women accessing HIV prevention services, female sex workers, men who have sex with men, pregnant women, and people living with HIV, have been reported to experience a higher burden of infection [24,25]. In our cohort, the syphilis prevalence of 4.9% surpasses the estimated prevalence for the Southern Africa region reported in a meta-analysis conducted in 2024 [26]. It is important to note that the syndromic management of STIs, introduced in the late 1990s alongside antenatal care screening, has contributed to a significant decline in syphilis prevalence in South Africa [25].

The co-infection rates in our cohort (0.1%) are considerably lower than those reported among women survivors of sexual violence in the Democratic Republic of Congo [20], as well as in other populations in South Africa [27,28,29]. The variation in prevalence could be due to differences in study populations and reporting periods, highlighting the evolving nature of the epidemics for these infections [20,21]. Notwithstanding these possible variations, the three infections share common risk factors, with exposure to one increasing the likelihood of contracting the others [30,31]. Therefore, it is crucial to screen all survivors of sexual violence for HIV, HBV, and syphilis, along with other STIs, both at the time of initial presentation and after the incubation period for each infection. This approach helps to identify infections that may have been acquired during the assault (depending on timeframes) or those that were present prior to the incident.

We found an association between age and both HIV and syphilis infections. Younger survivors (under 25 years of age) were less likely to be infected with these diseases than those over the age of 25. A similar association between age and HIV and syphilis infections has been reported in various populations across sub-Sahara African [20,27,32,33]. For example, a cohort study of pregnant women attending an antenatal clinic at KwaZulu-Natal Province, South Africa, found that older pregnant women had a higher risk of HIV and syphilis than younger pregnant women [27]. Similarly, a study in Rwanda reported that the prevalence of syphilis increased with age, rising from 0.6% in younger age groups to 1.1% in older individuals [32]. In a related study examining the prevalence of HBV and HIV among female survivors of sexual violence in the Democratic Republic of Congo, women aged 35 years or older were 1.8 times more likely to have HBV infection [20]. However, we did not explore the risk factors for HBV in our cohort owing to the relatively low number of confirmed cases.

We identified no cases of syphilis infection among survivors under the age of 12, but two and seven cases of HBV and HIV infections, respectively, were recorded in this age group. This suggests that individuals in this age group may not be sexually active enough to have acquired syphilis and other common STIs, but they could have contracted HIV and HBV through other routes, especially vertical transmission. In addition, we observed that survivors residing in urban areas were less likely to test positive for syphilis infection than those residing in peri-urban or rural areas. While the reason for this finding is not clear, it is possible that urban residents have better access to STI services than those living in the rural South African settings [34].

The lockdowns and other restrictive measures implemented in South Africa to control the COVID-19 pandemic may have trapped many survivors with their abusers, potentially increasing cases of sexual violence and the spread of HIV and other STIs [35]. However, our findings indicate that the number of reported cases of HIV, syphilis, and HBV in our cohort was higher before the pandemic than during it. Lockdowns, movement restrictions, and fear of contracting COVID-19 may have discouraged survivors from seeking healthcare, resulting in underreporting and decreased attendance at the treatment centres. Additionally, disruptions in healthcare services may have contributed to fewer recorded diagnoses. Social and economic instability during the pandemic may have further influenced reporting patterns, as survivors faced barriers such as transportation difficulties and safety concerns when seeking medical care. Therefore, the lower number of recorded cases during the pandemic likely reflects reduced access to and use of healthcare services rather than an actual decline in infections.

One limitation of our study was its retrospective design, which precluded considerations of unrecorded confounders or other potential risk factors, such as the number of sexual partners (concurrent or sequential) and prior sexual violence experiences, which were not captured in our database. Our rates may have underestimated or overestimated the prevalence of HBV, HIV, and syphilis in the province, as our analysis was restricted to survivors who attended the selected healthcare facilities. Some survivors of sexual violence, particularly males, may have avoided seeking care at the health centres because of fear of stigma, shame, lack of trust in healthcare professionals, or logistical challenges [10,11,36]. Furthermore, HBsAg was the only available measure of HBV infection, preventing differentiation between acute and chronic infections. In addition, occult HBV infection (detectable HBV viral load with negative HBsAg) may have been overlooked [37]. A large-scale, prospective, longitudinal, multi-site study that captures diverse demographic and socio-economic variables could provide further insights into the true epidemiology of these preventable infections among survivors of sexual violence in South Africa.

## 5. Conclusions

HBV, HIV, and syphilis infections are prevalent among male and female survivors of sexual violence, particularly those who are older or reside in non-urban areas. These findings underscore the substantial health burden faced by this vulnerable population and emphasise the need for integrated interventions that address both the physical and emotional consequences of sexual violence. Comprehensive screening and treatment protocols should be prioritised, along with improved access to post-exposure prophylaxis, vaccination initiatives, and sexual health education. By addressing these healthcare gaps, we can better support survivors, reduce the long-term impact of STIs within this population, and curb further transmission within the broader community.

## Figures and Tables

**Table 1:** Sociodemographic characteristics and seroprevalence of hepatitis B (HBV), human immunodeficiency virus (HIV), and syphilis among 1957 survivors of sexual violence.

Variable	Total	HIV			Syphilis			HBV		
		Negative [*n* (%)]	Positive [*n* (%)]	NS [*n* (%)]	Negative [*n* (%)]	Positive [*n* (%)]	NS [*n* (%)]	Negative [*n* (%)]	Positive [*n* (%)]	NS [*n* (%)]
All	1957 (100)	1251 (63.9)	338 (17.3)	368 (18.8)	1405 (71.8)	96 (4.9)	456 (23.3)	1504 (76.9)	14 (0.7)	439 (22.4)
Age (years), mean ± SD	21.66 ± 16.06	21.1 ± 16.3	29.3 ± 11.4	16.4 ± 14.1	22.4 ± 14.9	37.6 ± 18.5	16.7 ± 15.5	22.9 ± 15.4	38.3 ± 22.5	18.3 ± 15.8
<12	452 (23.1)	281 (22.5)	7 (2.1)	164 (44.6)	241 (17.2)	0 (0.0)	211 (46.3)	251 (16.7)	2 (14.3)	199 (45.3)
12–16	428 (21.9)	344 (27.5)	18 (5.3)	66 (17.9)	341 (24.3)	4 (4.2)	83 (18.2)	345 (22.9)	1 (7.1)	82 (18.7)
17–25	533 (27.2)	349 (27.9)	121 (35.8)	63 (17.1)	433 (30.8)	28 (29.2)	72 (15.8)	464 (30.9)	1 (7.1)	68 (15.5)
>25	541 (27.6)	275 (22.0)	192 (56.8)	74 (20.1)	388 (27.6)	64 (66.7)	89 (19.5)	442 (29.4)	10 (71.4)	89 (20.3)
Sex										
Male	125 (6.4)	85 (6.8)	10 (3.0)	30 (8.2)	86 (6.1)	5 (5.2)	34 (7.5)	91 (6.1)	1 (7.1)	33 (7.5)
Female	1832 (93.6)	1166 (93.2)	328 (97.0)	338 (91.8)	1319 (93.9)	91 (94.8)	422 (92.5)	1413 (93.9)	13 (92.9)	406 (92.5)
Race										
Black	1884 (96.3)	1211 (96.8)	336 (99.4)	337 (91.6)	1369 (97.4)	93 (96.9)	422 (92.5)	1464 (97.3)	14 (100.0)	406 (92.5)
White	24 (1.2)	13 (1.0)	0 (0.0)	11 (3.0)	10 (0.7)	2 (2.1)	12 (2.6)	13 (0.9)	0 (0.0)	11 (2.5)
Other	48 (2.5)	26 (2.1)	2 (0.6)	20 (5.4)	25 (1.8)	1 (1.0)	22 (4.8)	26 (1.7)	0 (0.0)	22 (5.0)
Residential area										
Rural	892 (45.6)	631 (50.4)	158 (46.7)	103 (28.0)	723 (51.5)	20 (20.8)	149 (32.7)	739 (49.1)	6 (42.9)	147 (33.5)
Peri-urban	892 (45.6)	515 (41.2)	157 (46.4)	220 (59.8)	571 (40.6)	64 (66.7)	257 (56.4)	642 (42.7)	6 (42.9)	224 (51.0)
Urban	172 (8.8)	105 (8.4)	23 (6.8)	44 (12.0)	111 (7.9)	12 (12.5)	49 (10.7)	123 (8.2)	2 (14.3)	47 (10.7)

Data are presented as mean ± standard deviation for continuous measures and *n* (%) for categorical measures. HBV, hepatitis B virus; HIV, human immunodeficiency virus; NS, not screened or missing; SD, standard deviation.

**Table 2:** Multivariate analysis of factors associated with human immunodeficiency virus (HIV) (*n* = 1589) and syphilis (*n* = 1501) infections among survivors of sexual violence.

Variable	HIV				Syphilis			
	OR (95% CI)	*p* Value	Adjusted OR (95% CI)	*p* Value	OR (95% CI)	*p* Value	Adjusted OR (95% CI)	*p* Value
Age (years)								
<16	0.04 (0.02–0.07)	<0.001	0.05 (0.03–0.08)	<0.001	0.05 (0.01–0.15)	<0.001	0.05 (0.02–0.15)	<0.001
16–25	0.45 (0.34–0.59)	0.009	0.45 (0.34–0.59)	0.008	0.45 (0.28–0.71)	<0.001	0.41 (0.25–0.66)	<0.001
>25	1.00	–	1.00	–	1.00	–	1.00	–
Sex								
Male	0.69 (0.33–1.40)	0.199	0.70 (0.34–1.45)	0.153	0.84 (0.33–2.12)	0.707	1.09 (0.41–2.90)	0.994
Female	1.00	–	1.00	–	1.00	–	1.00	–
Race								
Black	5.41 (1.30–22.52)	0.020	4.78 (1.09–20.88)	0.047	0.79 (0.23–2.62)	0.704	0.89 (0.25–3.17)	0.776
Other	1.00	–	1.00	–	1.00	–	1.00	–
Residential area								
Urban	1.17 (0.70–1.97)	0.312	1.10 (0.65–1.85)	0.251	0.25 (0.12–0.54)	<0.001	0.23 (0.10–0.50)	<0.001
Peri-urban	1.28 (0.76–2.15)	0.247	1.25 (0.74–2.11)	0.722	1.04 (0.54–1.98)	0.912	0.91 (0.46–1.80)	0.821
Rural	1.00	–	1.00	–	1.00	–	1.00	–

CI, confidence interval; HIV, human immunodeficiency virus; OR, odds ratio.

**Table 3:** Distribution of human immunodeficiency virus (HIV), syphilis, and hepatitis B (HBV) cases before and during the COVID-19 pandemic, categorised by lockdown restriction levels.

	HIV (*n* = 338)	Syphilis (*n* = 96)	HBV (*n* = 14)
Pre-COVID-19	234	68	11
During COVID-19	104	28	3
Alert Level 0	5	2	0
Alert Level 1	56	13	3
Alert Level 2	7	5	0
Alert Level 3	19	4	0
Alert Level 4	8	2	0
Alert Level 5	9	2	0

COVID-19, coronavirus disease 2019; HBV, hepatitis B virus; HIV, human immunodeficiency virus.

## Data Availability

The raw data supporting the conclusions of this article will be made available by the authors on request.

## Abbreviations

The following abbreviations are used in this manuscript:

aOR	Adjusted odds ratio
CI	Confidence interval
ELISA	Enzyme-linked immunosorbent assay
HBsAg	Hepatitis B surface antigen
HBV	Hepatitis B virus
HIV	Human immunodeficiency virus
MCAR	Missing Completely at Random
RPR	Rapid plasma reagin
SD	Standard deviation
STI	Sexually transmitted infection
TPHA	Treponema pallidum haemagglutination assay

## Appendix A

**Table A1:** Sensitivity analysis for the study variables.

Variable	HIV			Syphilis			HBV		
	With Results [*n* (%)]	No Results [*n* (%)]	*p* Value	With Results [*n* (%)]	No Results [*n* (%)]	*p* Value	With Results [*n* (%)]	No Results [*n* (%)]	*p* Value
All	1598	368		1501	456		1518	439	
Age (years)			<0.001			<0.001			<0.001
<16	650 (40.7)	230 (62.5)		586 (39.0)	294 (64.5)		599 (39.5)	281 (64.0)	
17–25	470 (29.40	63 (17.1)		461 (30.7)	72 (15.8)		465 (30.6)	68 (15.5)	
>25	469 (29.3)	75 (20.4)		454 (30.2)	90 (19.7)		454 (29.9)	90 (20.5)	
Sex			0.124			0.287			0.269
Male	95 (5.9)	30 (8.2)		91 (6.1)	34 (7.5)		92 (6.1)	33 (7.5)	
Female	1494 (93.5)	338 (91.8)		1410 (93.9)	422 (92.5)		1426 (93.9)	406 (92.5)	
Race			<0.001			<0.001			0.001
Black	1547 (96.8)	337 (91.6)		1462 (97.4)	422 (92.5)		1478 (97.4)	406 (92.5)	
White	13 (0.8)	11 (3.0)		12 (0.8)	12 (2.6)		13 (0.9)	11 (2.5)	
Other	28 (1.8)	20 (5.4)		26 (1.7)	22 (4.8)		26 (1.7)	22 (5.0)	
Residential area			<0.001			0.001			<0.001
Rural	789 (49.4)	103 (28.0)		743 (49.5)	149 (32.7)		745 (49.1)	147 (33.5)	
Semi-urban	672 (42.1)	220 (59.8)		635 (42.3)	257 (56.4)		648 (42.7)	244 (55.6)	
Urban	128 (8.0)	44 (12.0)		123 (8.2)	49 (10.7)		125 (8.2)	47 (10.7)	

Data are presented as *n* (%) unless otherwise indicated. HBV, hepatitis B virus; HIV, human immunodeficiency virus.
